# Skp1 Independent Function of Cdc53/Cul1 in F-box Protein Homeostasis

**DOI:** 10.1371/journal.pgen.1005727

**Published:** 2015-12-10

**Authors:** Radhika Mathur, James L. Yen, Peter Kaiser

**Affiliations:** Department of Biological Chemistry, College of Medicine, University of California Irvine, Irvine, California, United States of America; University of California San Francisco, UNITED STATES

## Abstract

Abundance of substrate receptor subunits of Cullin-RING ubiquitin ligases (CRLs) is tightly controlled to maintain the full repertoire of CRLs. Unbalanced levels can lead to sequestration of CRL core components by a few overabundant substrate receptors. Numerous diseases, including cancer, have been associated with misregulation of substrate receptor components, particularly for the largest class of CRLs, the SCF ligases. One relevant mechanism that controls abundance of their substrate receptors, the F-box proteins, is autocatalytic ubiquitylation by intact SCF complex followed by proteasome-mediated degradation. Here we describe an additional pathway for regulation of F-box proteins on the example of yeast Met30. This ubiquitylation and degradation pathway acts on Met30 that is dissociated from Skp1. Unexpectedly, this pathway required the cullin component Cdc53/Cul1 but was independent of the other central SCF component Skp1. We demonstrated that this non-canonical degradation pathway is critical for chromosome stability and effective defense against heavy metal stress. More importantly, our results assign important biological functions to a sub-complex of cullin-RING ligases that comprises Cdc53/Rbx1/Cdc34, but is independent of Skp1.

## Author Summary

Protein ubiquitylation is the covalent attachment of the small protein ubiquitin onto other proteins and is a key regulatory pathway for most biological processes. The central components of the ubiquitylation process are the E3 ligases, which recognize substrate proteins. The best-studied E3 complexes are the SCF ligases, which are composed of 3 core components—Cdc53, Skp1, Rbx1—that assemble to the functional ligase complex by binding to one of the multiple substrate adaptors—the F-box proteins. Maintaining a balanced repertoire of diverse SCF complexes that represent the entire cellular panel of substrate adapters is challenging. Depending on the cell type, hundreds of different F-box proteins can compete for the single binding site on the common SCF core complex. Rapid degradation of F-box proteins helps in maintaining a critical level of unoccupied Cdc53/Skp1/Rbx1 core, complexes and alterations in levels of F-box proteins has been linked to diseases including cancer. Studying the yeast F-box protein Met30 as a model, we have uncovered a novel mechanism for degradation of F-box proteins. This pathway targets free F-box proteins and requires part of the SCF core. These findings add an additional layer to our understanding of regulation of multisubunit E3 ligase.

## Introduction

Ubiquitin dependent proteolysis controls many cellular processes including signal transduction and cell cycle progression. Ubiquitin is covalently linked to substrates in a multistep process that requires coordinated action of 3 classes of enzymes- E1 ubiquitin activating enzyme, E2 ubiquitin conjugating enzyme, and E3 ubiquitin ligase [1–5]. E3 ubiquitin ligases are the key players in this system as they mediate substrate specific covalent attachment of ubiquitin. Within the E3 ligase family, cullin-RING ligases (CRLs) comprise the largest class, and in this group the SCF ubiquitin ligases are one of the best-understood complexes [2,6]. They are composed of yeast Cdc53 (mammalian cullin-1), Skp1, Rbx1, and one of the multiple F-box proteins, which bind substrates and confer specificity to the complex [7,8].

Amongst the SCF components, F-box proteins are relatively unstable in nature, which contributes to the dynamic assembly of a diverse repertoire of SCF complexes within the cell [9–13]. Accordingly, over expression of a single F-box protein in yeast can change the balanced distribution and diversity of available SCF complexes by sequestering cullin and Skp1 and thus block formation of functional SCF complexes with other F-box proteins [10,11,14]. Many F-box proteins control degradation of critical oncogenes and tumor suppressors and variation in their abundance has been linked to cancer [15,16]. Thus, it is important to understand how cells maintain F-box protein homeostasis. F-box proteins are known to be regulated by autoubiquitylation where their degradation is dependent upon their incorporation into a functional SCF complex [10,11]. The autocatalytic F-box protein degradation pathway is thought to be suppressed by substrate binding resulting in coordination of substrate availability with abundance of the corresponding assembled SCF complex [17,18]. Additional degradation pathways for F-box proteins are likely as it is also important to restrict abundance of unbound F-box proteins to prevent substrate shielding effects that would compete with substrate recognition by fully assembled ligases. Indeed, mammalian Skp2 is targeted for degradation by the anaphase promoting complex or cyclosome [19,20], Fbx5 (Emi1) is degraded by SCF^βTrCP/Slimb^ [21], and the level of the budding yeast F-box protein Dia2, which is required for genomic stability, is restricted by the HECT domain E3 ligase Tom1 [22].

In *Saccharomyces cerevisiae*, Met30 is one of three essential F-box proteins. SCF^Met30^ coordinates metabolic pathways of sulfur containing compounds with cell cycle progression. The transcription factor Met4 is a key target of SCF^Met30^ [23,24]. Low levels of the methyl donor, S-adenosylmethionine cause a block in SCF^Met30^ dependent ubiquitylation of Met4, activating it and results in cell cycle arrest and transcription of methionine response genes [25]. SCF^Met30^ also represses expression of enzymes responsible for glutathione synthesis and is thus a key factor in response to heavy metal stress. Cadmium exposure induces active dissociation of Met30 from Skp1, thereby inhibiting SCF^Met30^ and inducing glutathione production and cell cycle arrest, which together protect cellular integrity [26–28]. Therefore, the SCF^Met30^ system generates unbound Met30 via heavy metal stress induced dissociation from Skp1. To prevent unwanted effects of excess unbound Met30, such as substrate sequestration, it seemed important, that a degradation mechanism exists in addition to autocatalysis that plays an integral role in maintaining Met30 homeostasis.

Here we report such an additional mechanism for Met30 regulation in addition to autoubiquitylation. This degradation pathway targets Met30 that is detached from Skp1 and involves the SCF core components Cdc53 and Rbx1, as well as the cognate SCF ubiquitin conjugating enzyme Cdc34. Importantly, this ubiquitylation pathway does not require Skp1 (Skp1-independent Cul1-dependent ubiquitylation) and suggests a function of Cdc53/Cul1 that is independent from association with Skp1.

## Results

### Dissociation of the F-box protein Met30 from Skp1 targets it for degradation

**Fig 1:**
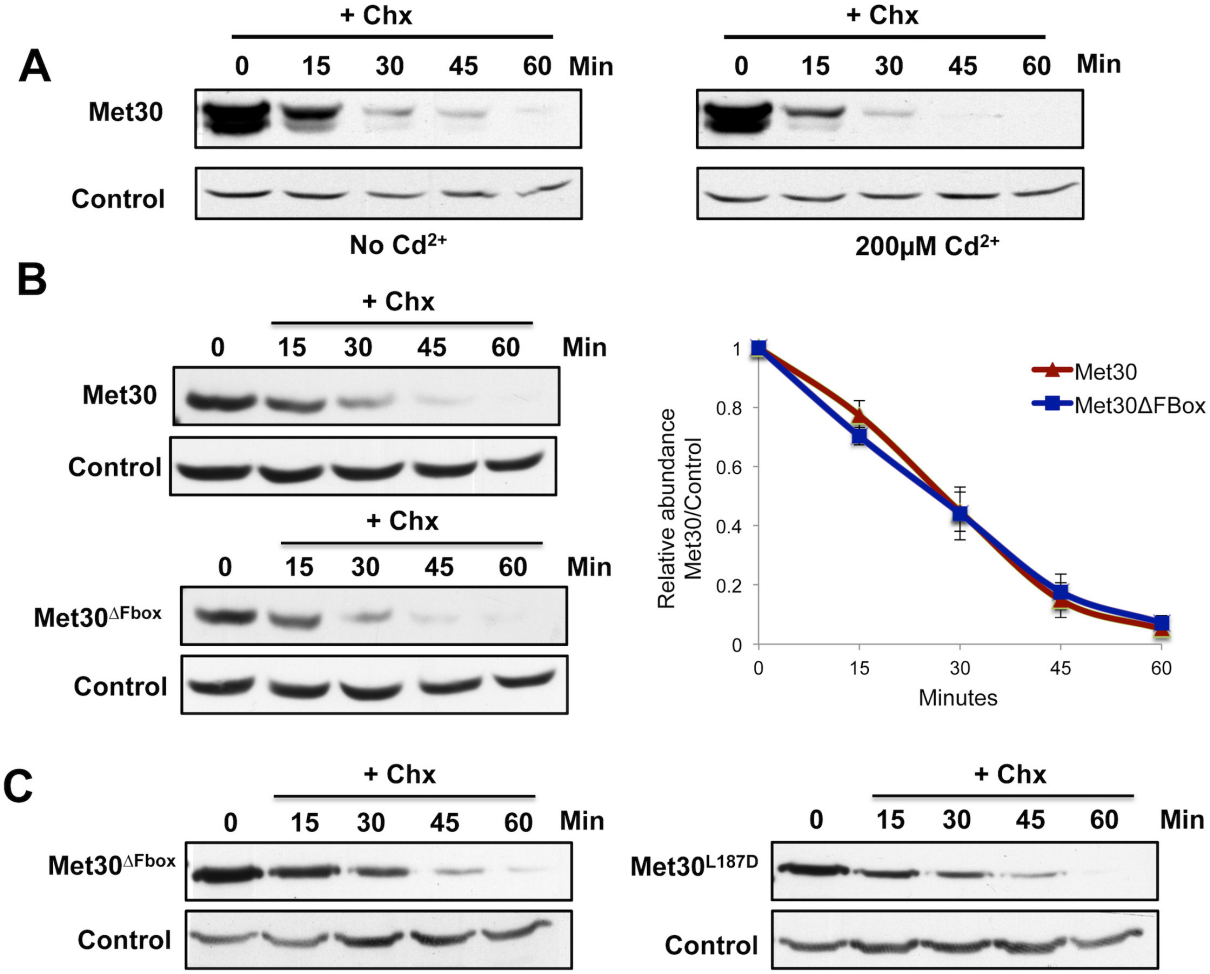
Met30 dissociated from Skp1 is rapidly degradation. (A) Cells expressing endogenous ^12Myc^Met30 were grown at 30°C. Cadmium was added to a final concentration of 200μM and protein translation was inhibited by addition of 100μg/ml cycloheximide. Samples were collected at the time intervals indicated and Met30 stability was analyzed by immunoblotting with anti-myc antibodies. The proteasome subunit, Cim5 was detected as a loading control. (B) Experiment as in panel A, but experiment was performed with cells expressing either endogenous ^12Myc^Met30 or ^12myc^Met30^ΔFbox^ (residues 187–227 deleted). Quantification was performed a Fuji LAS-4000 imaging system followed by analyses with the Multi Gauge v3 software. Results are presented as mean ± standard error for three independent experiments (right panel) (C) Experiment as described for panel A, cells expressing endogenous ^12Myc^Met30^ΔFbox^ and ^12Myc^Met30^L187D^ were compared.

Defense against heavy metal toxicity requires coordinated changes of metabolic pathway flux connected to glutathione synthesis, induction of a cell cycle checkpoint response to avoid continued cell division during stress conditions, and cell protective measures known as sulfur sparing [25]. SCF^Met30^ is the key regulator of this concerted response [24,26]. Coordinated regulation is achieved because cadmium stress disrupts ubiquitylation of all SCF^Met30^ substrates by the active and selective disassembly of the SCF^Met30^ protein complex, but not other SCF ligases [24,26,27]. This is accomplished by Cdc48-mediated dissociation of the F-box component Met30 from Skp1 [24,26,27], which results in generation of a pool of unbound Met30. In addition, Met30 transcription is induced by over seven fold during cadmium stress [29], which further increases the amount of Met30 that is not associated with core SCF components (referred to as unbound or ‘Skp1-free’ Met30). We were interested in how cells cope with the generated excess of Met30 because the well-described autocatalytic F-box protein degradation pathway mechanism [10,11] cannot act on Met30 during these conditions. Interestingly, degradation of the F-box protein Met30 was maintained and even slightly induced in response to cadmium stress (Fig 1A). We next asked whether cadmium stress induces a degradation pathway for unbound Met30, or if dissociation of Met30 from Skp1 might be sufficient to induce its degradation. To this end we examined stability of Met30 that cannot associate with Skp1 even in the absence of cadmium due to mutations in the F-box motif, which forms the Skp1 interaction surface. A mutant form of Met30 lacking its entire F-box domain (Met30^ΔFbox^) was constitutively unstable even though cells did not experience cadmium stress (Fig 1B). Rapid degradation of Met30^ΔFbox^ was observed both in cycloheximide chase and promoter shut-off experiments, indicating that components of this degradation pathway are constitutively present and that new protein synthesis is not required (S1A Fig). Disruption of the Met30-Skp1 interaction by the single amino acid change L187D [30], rather than complete deletion of the F-box domain, was sufficient to induce Met30 degradation (Figs 1C and S1B) suggesting that Met30 that is not bound to Skp1 is degraded.

To further test this idea and exclude that mutating the F-box region results in conformational changes that target Met30 to a non-physiological degradation pathway, we measured Met30 stability in another condition where Met30 is dissociated from Skp1. To this end we used a yeast strain carrying the temperature sensitive *skp1-*25 allele, which is inactivated by a temperature shift and disrupts the integrity of SCF ligases at the restrictive temperature [31]. Met30 was efficiently degraded in *skp1-25* mutants and cadmium exposure did not further destabilize Met30 (S1C Fig).

Collectively these results demonstrate that disruption of the Met30-Skp1 interaction, by either active signal-induced dissociation (cadmium stress) or by mutations in Met30 or Skp1, induces rapid degradation of the F-box protein Met30. These results suggest a proteolytic pathway that recognizes unbound ‘Skp1-free’ Met30 to avoid accumulation of excess unbound Met30, which could bind substrates and shield them from recognition by fully assembled SCF^Met30^ complexes.

### Degradation of ‘Skp1-free’ Met30 requires Cdc53, Cdc34, and Rbx1 but not Skp1

**Fig 2:**
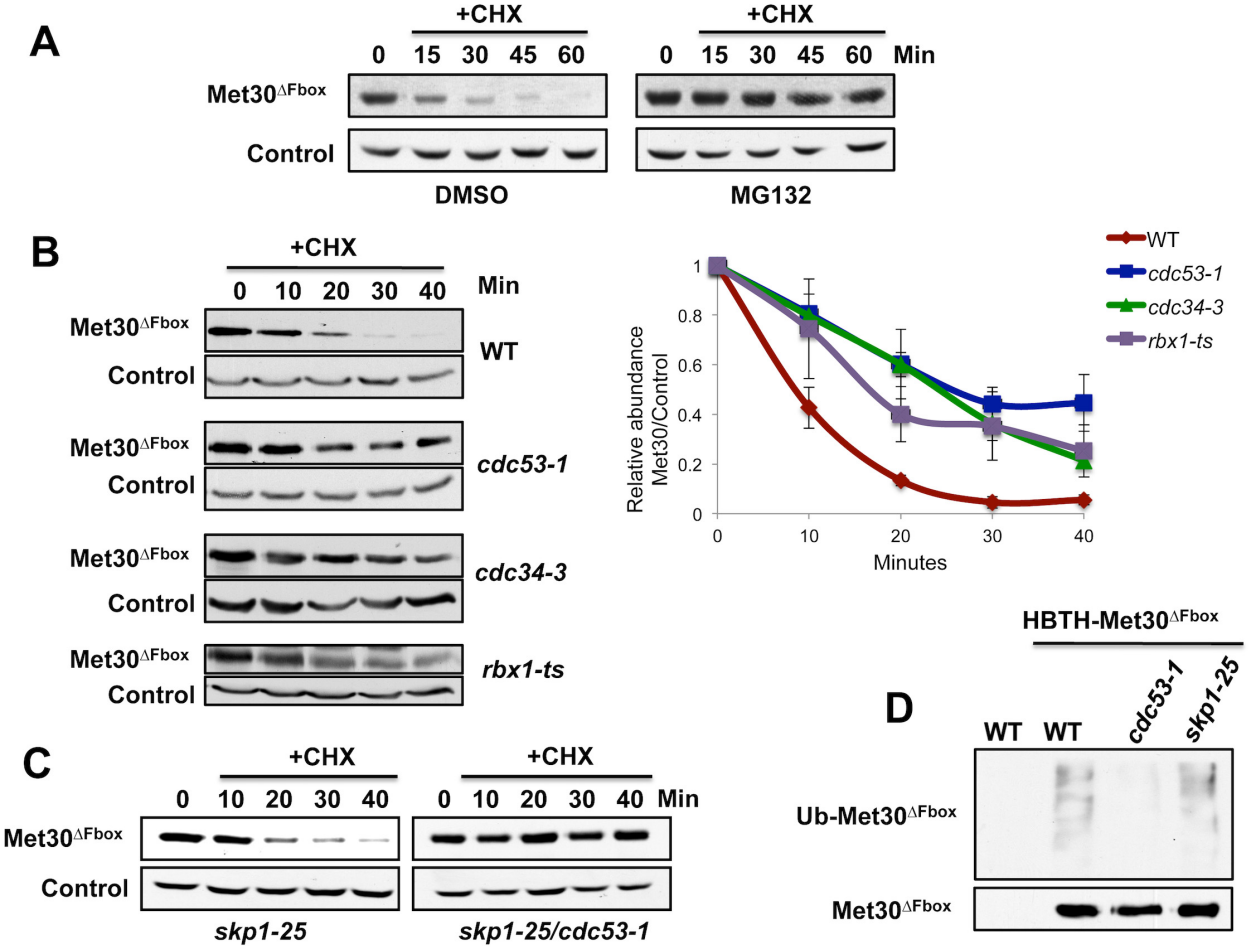
Degradation of ‘Skp1-free’ Met30 depends on the proteasome, Cdc53, Cdc34, and Rbx1, but is independent of Skp1. (A) Cells expressing either endogenous ^12Myc^Met30 or ^12myc^Met30^ΔFbox^ were grown at 30°C. Proteasomes were inhibited with 50μM MG-132 for 45 min before cycloheximide was added to block translation. Cells carried a deletion of *PDR5* to increase MG-132 permeability. (B) ^12Myc^Met30^ΔFbox^ stability was analyzed in wild type, *cdc34-3*, and *cdc53-1* and *rbx1-13myc* temperature sensitive mutants by cycloheximide chase experiments and immunoblotting with anti-myc antibodies as described for Fig 1A. Cells were grown at 25°C, shifted to 37°C for 1.5 h to inactivate temperature sensitive mutants (C) Experiment as in panel B, but ^12myc^Met30^ΔFbox^ stability was analyzed in *skp1-25* single and *skp1-25 cdc53-1* double mutants. (D) Cells expressing ^HBTH^Met30^ΔFbox^ under control of the *GAL1* promoter were shifted to 37°C for 1.5 h to inactivate temperature sensitive alleles. ^HBTH^Met30^ΔFbox^ was purified on Ni^2+^-sepharose under denaturing conditions and analyzed by immunoblotting using antibodies directed against ubiquitin (upper panel) or the RGS6H epitope in the HBTH tag (lower panel). Cells expressing untagged Met30^ΔFbox^ were processed as control.

We sought to further characterize proteolysis of ‘Skp1-free’ or unbound Met30 and asked whether it was dependent upon the ubiquitin-proteasome system. In wild-type cells Met30 may exist either bound or unbound to the SCF core. In order to specifically study the degradation pathway targeting ‘Skp1-free’ Met30, we used Met30^ΔFbox^ as a tool to generate a homogenous population of Met30 free from Skp1. Inhibition of proteasome activity with MG-132 led to stabilization of Met30^ΔFbox^, suggesting that the ubiquitin proteasome pathway is involved in degradation of ‘Skp1-free’ Met30 (Fig 2A). We next asked what E2 ubiquitin conjugating enzyme might be involved in this degradation process. Surprisingly, degradation of Met30^ΔFbox^ was dependent on the canonical SCF E2, Cdc34 (Fig 2B). The requirement for Cdc34 compelled us to test dependence on SCF core-components even though our previous experiments demonstrated that Skp1 is not involved in this degradation pathway. Unexpectedly, Cdc53 was indispensable for Met30^ΔFbox^ degradation, because inactivation of the temperature sensitive *cdc53-1* allele blocked degradation (Fig 2B). Consistent with these results Met30^ΔFbox^ degradation was greatly reduced in a strain expressing a 13Myc-tagged version of the RING finger component Rbx1, which has previously been shown to be a hypomorph allele that results in reduced SCF function at high temperature [32] (Fig 2B). These experiments not only suggest a degradation pathway for Met30 that is independent of the canonical autoubiquitylation mechanism, but more surprisingly, indicate that the cullin-1 (Cdc53) based ligase complex might have functions independent of its adaptor component Skp1. Given these unexpected results we wanted to further test this hypothesis and ensure that *skp1-25* and *cdc53-1* mutants probe the same degradation pathway and do not induce any secondary effects that might affect interpretation of the degradation results. We thus tested Met30^ΔFbox^ half-life in *skp1* single and *skp1 cdc53* double mutants. As previously observed, unbound Met30 was rapidly degraded in *skp1-25* mutants but, importantly, was stabilized upon inactivation of *cdc53-1* in the double mutant (Fig 2C), confirming that degradation was dependent on Cdc53 but not Skp1. In agreement with the Met30^ΔFbox^ degradation data, ubiquitylated Met30^ΔFbox^ was readily detectable in *skp1* mutants but absent in *cdc53* mutants further supporting the hypothesis of a Skp1- independent degradation function for Cdc53 (Fig 2D).

In addition of being a SCF^Met30^ substrate itself, the transcription factor Met4 has been shown to function as a substrate receptor in the context of SCF^Met30/Met4^ to coordinate degradation of its own co-factors [33]. Analysis of Met30^ΔFbox^ degradation in *met4Δ* mutants showed that it was not involved in degradation of ‘Skp1-free’ Met30 (S2A Fig).

Cullins are regulated by covalent modification with ubiquitin like protein Nedd8 (Rub1 in yeast), which induces a conformational rearrangement of cullin and stimulates ubiquitin transfer by the SCF-bound E2 [6]. Deneddylated cullins interact with Cand1 (Lag2 in yeast), which inhibits binding of Skp1- F-box protein complex and prevents SCF ligase function [12,34,35]. As rubylation is dispensable in yeast [36], we posited that Lag2 bound to Cdc53 may serve as an adaptor for regulating F-box proteins dissociated from Skp1. Deletion of *LAG2* failed to stabilize Met30^ΔFbox^ demonstrating that degradation of ‘Skp1-free’ Met30 is independent of Lag2 (S2B Fig).

Together these results suggest existence of a novel mechanism of Met30 regulation that specifically targets Met30 that is displaced from Skp1 and is dependent on the ubiquitin proteasome system and requires the function of Cdc53, Rbx1 and Cdc34, but not Skp1.

### Met30^ΔFbox^ degradation is independent of Skp1

**Fig 3:**
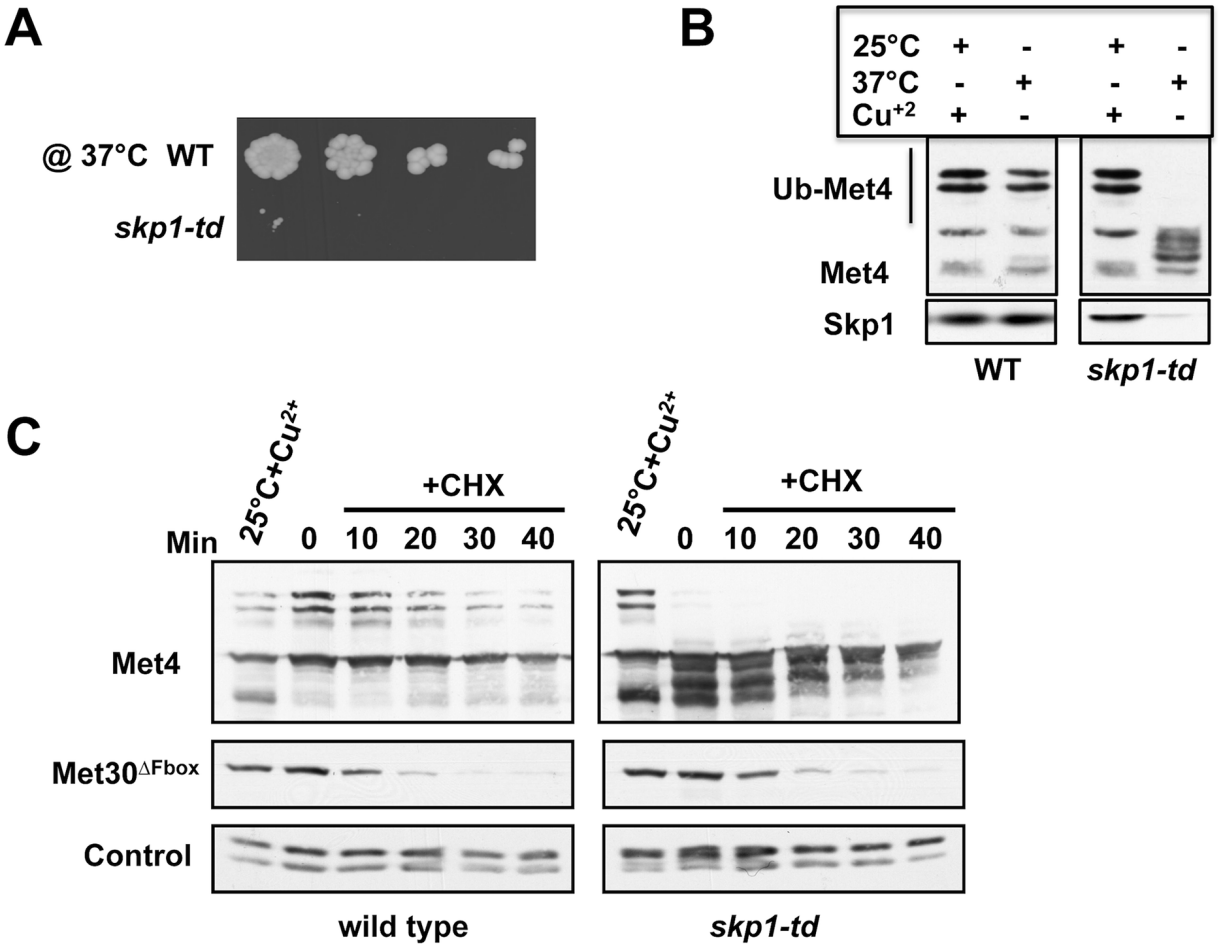
Skp1 independent degradation of Met30. (A) *skp1-td* mutants express an N-terminal temperature inducible degron (td) fused to Skp1 under the control of the *CUP1* promoter. Wild type and *skp1-td* cells were serially diluted and spotted on YEPD plates without copper at 37°C for 2 days. (B) Wild type and *skp1-td* mutants were cultured at permissive (25°C + Cu^2+^) and non-permissive conditions (37°C for 1 h without Cu^2+^). Immunoblot analysis showed that Met4 ubiquitylation was blocked in *skp1-td* mutants indicative of inactivation of SCF^Met30^. (C) Wild type and *skp1-td* strains expressing endogenous ^12myc^Met30^ΔFbox^ were cultured under permissive conditions and then shifted to non-permissive conditions for 1 h to deplete Skp1-td. Met30^ΔFbox^ stability was analyzed using cycloheximide to block translation. Immunoblot analysis of Met4 confirmed inactivation of Skp1-td. Anti-PSTAIR detection of Cdc28 was used as loading control.

Temperature sensitive *skp1* alleles have been previously shown to differentially affect SCF ligases depending on the identity of F-box protein subunits [10,37]. Although the *skp1-25* allele was specifically selected to represent a complete loss of Skp1 function, including inactivation of SCF^Cdc4^ and SCF^Met30^, we could not unambiguously exclude that Skp1-25 forms an intact SCF ligase that could ubiquitylate Met30^ΔFbox^ in trans. To address this issue we employed the temperature inducible degron tag (*td*) strategy to deplete Skp1 protein from cells [38], rather than rely on inactivation of temperature sensitive alleles. A *skp1-td* strain was constructed by expressing Skp1 fused to the temperature inducible degron under control of the inducible *CUP1* promoter. Attenuation of Skp1 induced the expected biological response such as cell cycle arrest with elongated multibudded cell morphology indicative of SCF^Cdc4^ inactivation, and block of SCF^Met30^ function as assayed by loss of ubiquitylated forms of Met4 (Fig 3A and 3B). Combined *CUP1* promoter repression and temperature induced Skp1-td thus efficiently ablated Skp1 function and protein level (Fig 3). Consistent with results using temperature sensitive alleles of *skp1*, Met30^ΔFbox^ degradation was unaffected when Skp1 function was blocked using the *skp1-td* strategy (Fig 3C). These results strongly support our hypothesis of Skp1-independent degradation of Met30.

### Cdc53 mutant unable to bind Skp1 can degrade Met30

**Fig 4:**
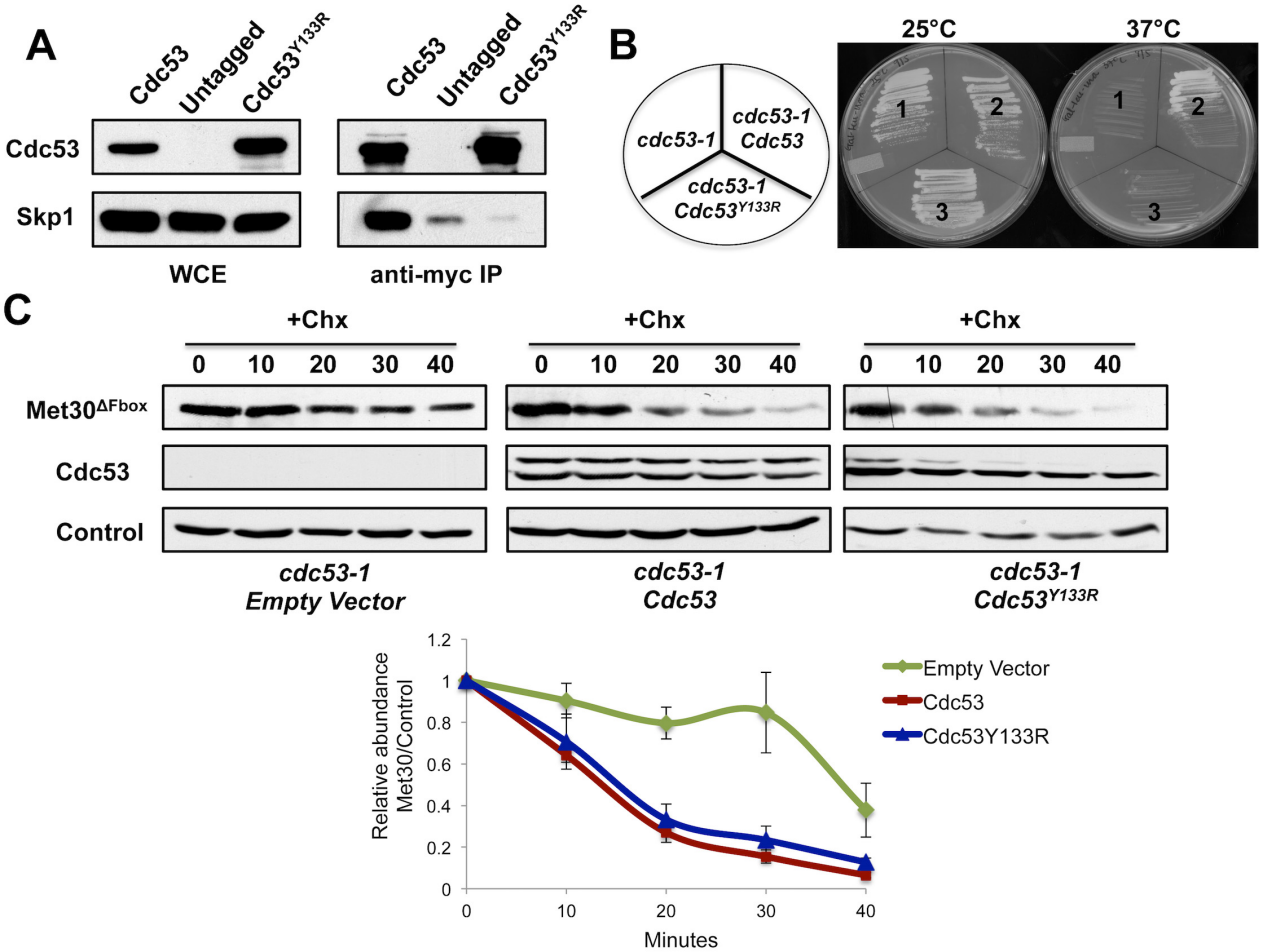
Cdc53 mutants unable to bind Skp1 can degrade Met30. (A) Cells expressing *GAL1* inducible ^12Myc^Cdc53 and ^12Myc^Cdc53^Y133R^ or empty vector control were grown at 30°C in medium containing 2% galactose to induce Cdc53 expression. ^12Myc^Cdc53 was immunopurified and co-purified proteins were analyzed by immunoblotting. WCE: Whole cell extract (B) A *cdc53-1* temperature sensitive strain containing Cdc53 expressing plasmids as indicated, were incubated on plates at 25°C or 37°C. (C) *cdc53-1* temperature sensitive mutants expressing ^12myc^Met30^ΔFbox^ under control of the native promoter and *GAL1* inducible RGS6H-tagged *CDC53* alleles as indicated or the empty vector control were grown at 25°C in medium containing 2% galactose to induce Cdc53 expression. Cultures were shifted to 37°C for 2 h to inactivate *cdc53-1* and ^12myc^Met30^ΔFbox^ stability was analyzed using cycloheximide to block translation. Quantification was performed as described for Fig 1. Results are presented as mean ± standard error for three independent experiments normalized to Cim5 (bottom panel).

The experiments with *skp1* single and *skp1 cdc53* double mutants strongly suggested that the ‘Skp1-free’ degradation pathway for Met30 was dependent on Cdc53 and independent of Skp1. We reasoned that in such a scenario, a Cdc53 mutant defective in interacting with Skp1 should be capable to degrade Met30^ΔFbox^. To examine this, a *GAL1* inducible Cdc53^Y133R^ mutant was constructed. Mutation of tyrosine in position 133 to arginine has previously been suggested to disrupt the Cdc53-Skp1 interaction [39]. Immunopurification experiments confirmed that Cdc53^Y133R^ does not interact with Skp1 *in vivo* (Fig 4A). Accordingly, expression of Cdc53^Y133R^ was unable to rescue the *cdc53-1* growth arrest phenotype at restrictive temperature (Fig 4B). Importantly, congruous with our hypothesis, Cdc53^Y133R^ supported degradation of Met30^ΔFbox^ to the same extent as wild type Cdc53 (Fig 4C). These results support the hypothesis of a cullin-1 (Cdc53) function in protein degradation independent of its adaptor component Skp1.

### Hydrophobic residues proximal to the F-box domain are required for degradation of ‘Skp1-free’ Met30

Instability of Met30 in *skp1* mutants led us to hypothesize that the ubiquitin ligase responsible for degrading ‘Skp1-free’ Met30 may recognize a domain close to the F-box domain, which is directly or indirectly obstructed by Skp1 binding. In accordance with this idea, a so-called R-motif has been described in the yeast F-box protein Cdc4, which is adjacent to its F-box domain. In addition, F-box—Skp1 interaction acts to suppress R-motif mediated Cdc4 degradation [14]. Thus, to identify the degradation sequences (degron) in Met30 recognized by the ‘Skp1-free’ degradation pathway, we generated deletions in Met30^ΔFbox^ near the F-box domain and compared their stability to that of Met30^ΔFbox^.

**Fig 5:**
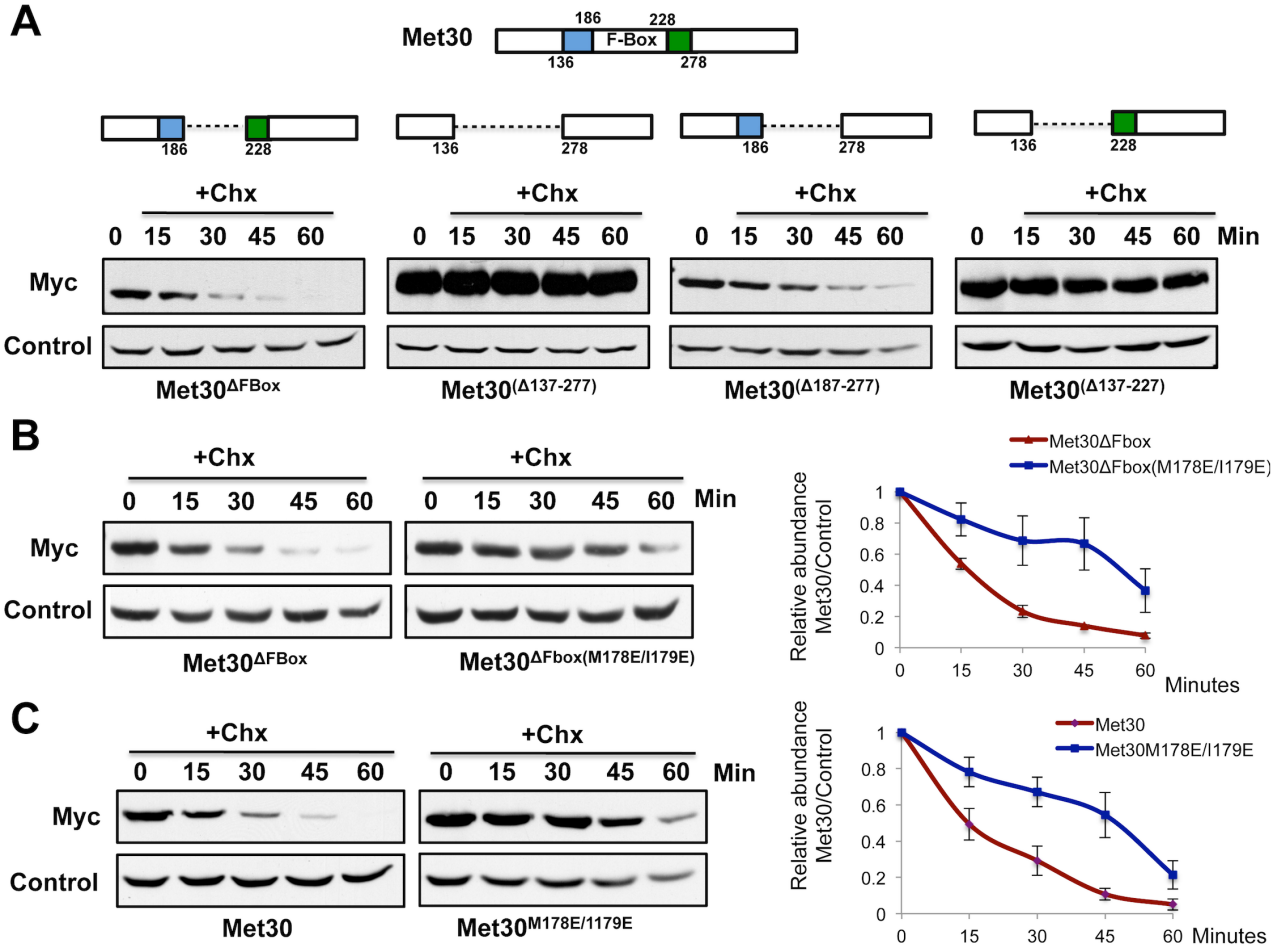
Mutation of methionine 178 and isoleucine 179 in Met30 abolishes ‘Skp1-free’ Met30 degradation. (A) Identification of a degron region for the ‘Skp1-free’ Met30 degradation pathway. Cells expressing either endogenous ^12myc^Met30^ΔFbox^ or different Met30^ΔFbox^ deletion mutants were grown at 30°C. Protein translation was inhibited by addition of cycloheximide and cells were collected at the time intervals indicated. Met30^ΔFbox^ stability was analyzed by immunoblotting with anti-myc antibodies. (B & C) Experiment as in panel A, with cells expressing endogenous ^12myc^Met30^ΔFbox^, full-length ^12myc^Met30, or the respective degron point mutants. Results are presented as mean ± standard error for three independent experiments (right panels).

Congruous with our hypothesis, deletion of 100 amino acids proximal and distal to the F-box domain (amino acids 137–277) not only increased steady-state Met30^ΔFbox^ levels (time point 0) but also completely prevented its degradation (Fig 5A). To further narrow down the degron sequence, shorter deletions encompassing either the C terminal or the N terminal 50 amino acids were constructed. Deletion of the N terminal amino acids adjacent to the F-box motif (amino acids 137–187) was sufficient for Met30^ΔFbox^ stabilization (Fig 5A). Interestingly, this region overlapped with a 45 amino acid stretch immediately N-terminal to the F-box domain, which mediates dimerization of WD-40 repeats containing F-box proteins and is conserved from yeast to humans [40]. Overlap of the degron region and the dimerization domain raised the possibility that the ligase for ‘Skp1-free’ Met30 degradation recognized the dimerization motif in Met30. However, mutation of isoleucine 159 and leucine 160, two amino acids crucial for Met30 dimerization [40], in Met30^ΔFbox^ failed to stabilize the protein (S3A Fig), suggesting that different residues within this stretch were being recognized by the ligase.

Smaller deletions within the N terminal 50 amino acids contiguous to the F-box domain suggested that amino acids 170–187 in Met30, corresponding to a region rich in hydrophobic residues, were important for ligase binding because various Met30^ΔFbox^ mutants containing this region were efficiently degraded and mutants lacking this region were stabilized (Figs 5A and S3B). To identify key residues in the degron, we mutated methionine 178 and isoleucine 179, a hydrophobic patch close to the F-box domain and conserved amongst WD-40 repeat containing F-box proteins. Mutation of both residues to glutamate (M178E and I179E) blocked ‘Skp1-free’ Met30^ΔFbox^ degradation (Fig 5B). Introduction of the same mutations into full length Met30 dramatically stabilized Met30 (Fig 5C), suggesting that under normal growth conditions the majority of Met30 in the cell was being targeted for proteolysis via the ‘Skp1-free’ degradation pathway.

### Cdc53/Rbx1 can bind Met30 in absence of Skp1

**Fig 6:**
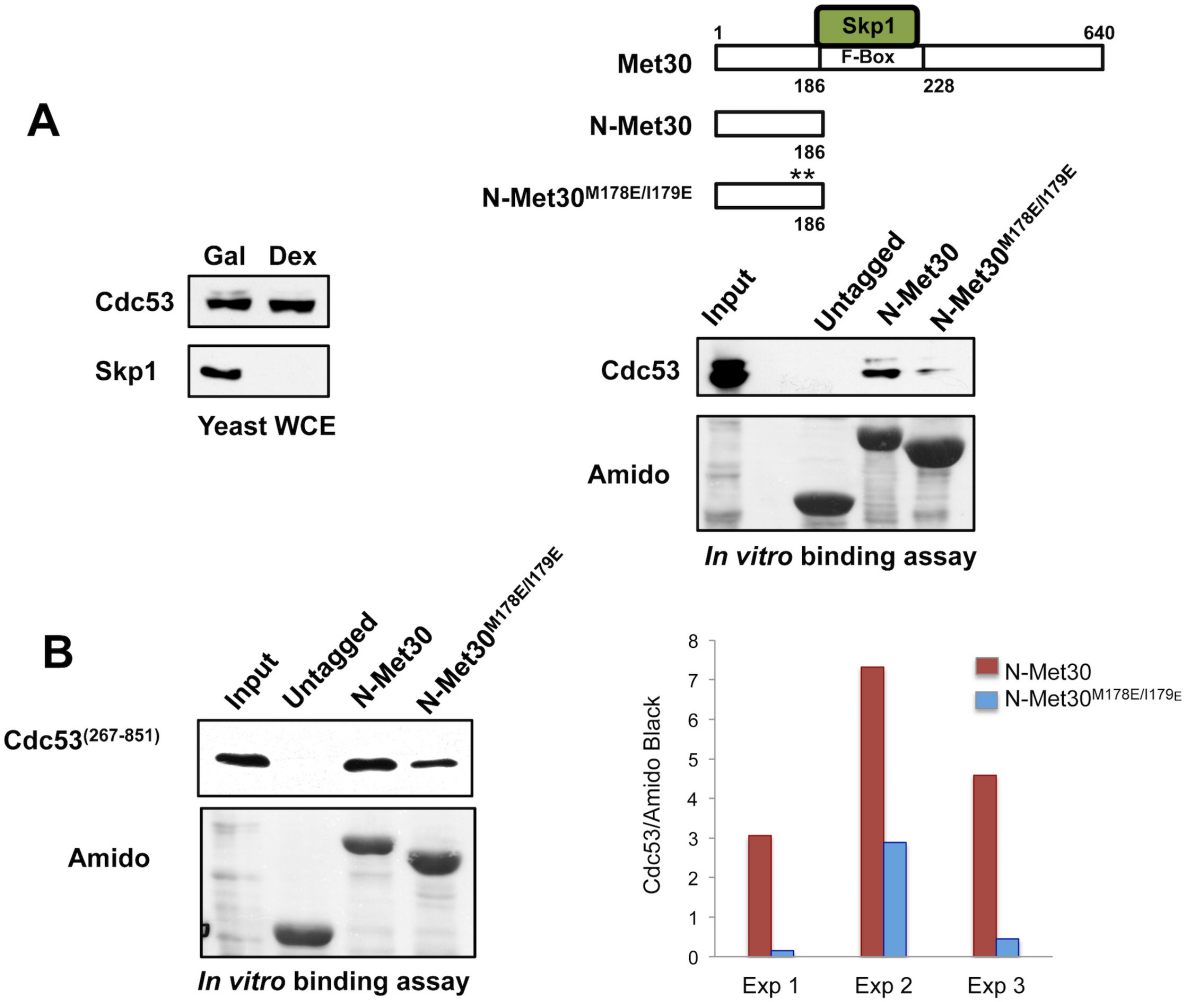
Cdc53/Rbx1 can bind Met30 in absence of Skp1. (A) Yeast strains expressing endogenous Cdc53^TAP^ and *GAL1* inducible Skp1 were cultured in media containing 2% galactose to express Skp1 and then shifted to media containing 2% dextrose for 12 h to deplete Skp1. Skp1 was efficiently depleted from cells (left panel). Schematic of the tagged Met30 constructs used for binding experiments (right top panel). MBP, (MBP)-Met30^(1–186)^ and (MBP)-Met30^(1–186)M178E/I179E^ were expressed in *E*.*coli* and bound to amylose resin. The resin was then incubated with Skp1 depleted yeast lysates expressing Cdc53^TAP^. Beads were washed and bound proteins were eluted and analyzed by Western blotting (lower panel on right). Cdc53 levels were detected with a PAP antibody recognizing the TAP tag. (B) Amylose beads were bound with MBP, (MBP)-Met30^(1–186)^ and (MBP)-Met30^(1–186)M178E/I179E^ and were incubated with bacterial lysate expressing Cdc53^267-851^/Rbx1. Beads were washed, bound proteins eluted and analyzed by Western blotting. Cdc53 levels were detected with anti-Cdc53 antibodies. Quantification was performed as described for Fig 1. Results are presented for three independent experiments normalized to the Amido black signal for MBP tagged proteins.

To determine the composition of the ligase and identify unknown adaptor components we performed mass spectrometry with purified Met30^ΔFbox^ to analyze its binding partners. In addition, we used the same strategy to profile Cdc53 interacting proteins, with the hope to identify adaptor proteins that function as Skp1 alternatives by searching for commonalities between these two mass spectrometry datasets. We failed to identify any proteins that fit these criteria. Although negative result cannot be conclusive, this result suggested that perhaps Cdc53/Rbx1 might directly bind Met30 without an adaptor protein. To examine binding in absence of Skp1, we used a yeast strain harboring a *GAL1-SKP1* allele such that Skp1 depletion could be achieved by growing the strain in media containing dextrose, which efficiently suppresses the *GAL1* promoter (Fig 6A, left panel). This yeast strain served as a source of protein extract lacking Skp1. Maltose-binding protein (MBP) fused to the N-terminal region of Met30 was expressed in bacteria and used as substrate. The *in vitro*-binding assay was performed with immobilized (MBP)-Met30^(1–186)^ and Skp1 depleted yeast lysates. In the absence of Skp1, Cdc53 bound effectively to (MBP)-Met30^(1–186)^ and binding was significantly reduced when (MBP)-Met30^(1–186)M178E/I179E^ was used as the bait (Fig 6A, right panel). Therefore, the same mutations, M178E and I179E that prevent degradation of Met30 by the ‘Skp1-free’ pathway *in vivo* also reduce Skp1-independent binding of Cdc53 to Met30 *in vitro*. Because this binding assay contained total yeast lysates, albeit lacking Skp1, it was possible that an unknown yeast protein mediated the interaction between (MBP)-Met30^(1–186)^ and Cdc53. To test whether any other factor apart from Cdc53/Rbx1 were necessary for this interaction, we utilized ^6XHis^Cdc53/^GST^Rbx1 expressed in bacteria [41] for *in vitro* binding experiment. Immobilized (MBP)-Met30^(1–186)^ was incubated with Cdc53/Rbx1 expressed in bacteria and binding was analyzed by immunoblotting with anti-Cdc53 antibodies. Similar to the result with yeast lysate, Cdc53/Rbx1 could specifically interact with (MBP)-Met30^(1–186)^ and binding was significantly decreased in the degron mutations that stabilized Met30 *in vivo* (Fig 6B). Cdc53 expressed in *E*. *coli* is spontaneously cleaved at the N-terminal region resulting in a truncated version (residues 267–851)[41]. Importantly, the removed residues 1–266 harbor the Skp1-binding region. Therefore, Met30 can interact with Cdc53/Rbx1 *in vitro* at a site distinct from the Skp1 binding domain of Cdc53.

As binding studies strongly suggested that Cdc53/Rbx1 could directly interact with Met30 without an adaptor protein, we tested whether Cdc53/Rbx1 could ubiquitylate ‘Skp1-free’ Met30 and was sufficient to function as a ligase. (MBP)-Met30^(1–186)^ was immobilized to amylose resin and incubated with bacterial lysate expressing Cdc53/Rbx1. The substrate-ligase complex was eluted and the ubiquitylation reaction was initiated by addition of the reaction mix containing E1 enzyme, E2 conjugating enzyme, ubiquitin and ATP. (MBP)-Met30^(1–186)^ was ubiquitylated *in vitro* and the reaction was dependent on Cdc53/Rbx1 complex (S4A Fig). However, the reaction is very inefficient and only a small fraction of Met30^(1–186)^ was ubiquitylated even after 16 h incubation of the reaction. To test whether this weak activity was specific for the ‘Skp1-free’ degradation pathway of Met30, we compared ubiquitylation in (MBP)-Met30^(1–186)^ and (MBP)-Met30^(1–186)M178E/I179E^ mutant. Ubiquitylation of (MBP)-Met30^(1–186)M178E/I179E^ was visibly reduced (S4B Fig). Although these experiments demonstrate ubiquitylation of Met30 by a minimal cullin-1 (Cdc53) complex lacking Skp1 the observed activity is not very robust and requires a long reaction time (~ 16 hours) and yet only a relatively small fraction of the substrate is ubiquitylated. This suggests possible involvement of another factor or a post-translational modification, which is likely not required for binding Cdc53/Rbx1 to Met30 but is required for effective ubiquitylation of the substrate. Alternatively, Cdc53 expressed in *E*. *coli* may not efficiently fold into its active structure and thus be only partially active.

### Skp1-independent degradation of F-box proteins Ctf13 and Cdc4

**Fig 7:**
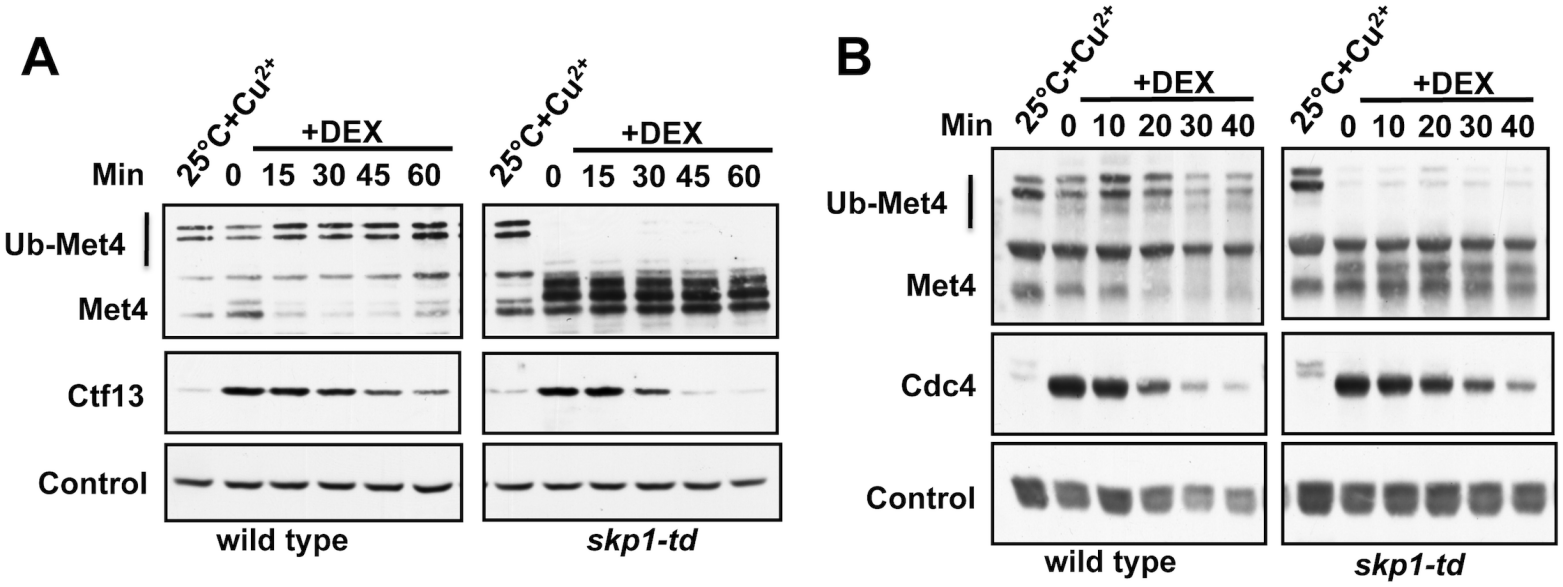
Skp1-independent degradation of Ctf13 and Cdc4. (A and B) *skp1-td* cells expressing ^13myc^Ctf13 or ^3myc^Cdc4 under control of the inducible *GAL1* promoter were depleted of Skp1-td as described for Fig 3C. Dextrose was added to terminate transcription from the *GAL1* promoter and degradation of Ctf13 and Cdc4 was analyzed by immunoblotting.

Our results strongly supported the hypothesis of Skp1-independent degradation of ‘free’ Met30. We next asked whether this Skp1-independent degradation pathway was unique for Met30 or may be a more general mechanism to limit F-box protein abundance. Such a mechanism for F-box protein homeostasis could be important not only to limit the overall abundance, but also to limit competition for substrates between assembled SCF ligases and the ‘free’ F-box protein subunits, which could lead to substrate shielding and thus prevent substrate ubiquitylation. Although degradation of the F-box protein Cdc4 has been demonstrated to follow the autoubiquitylation pathway [10,11] evidence has also been reported that Cdc4 continues to be degraded in *skp1-3* temperature sensitive mutants, and Cdc4 was also shown to be stabilized when Skp1 is overexpressed [14]. In addition, the kinetochore component and F-box protein Ctf13 was reported to be rapidly degraded in a Cdc34 dependent mechanism when Skp1 was inactivated [42]. These examples are consistent with ‘Skp1-free’ Cdc4 and Ctf13 degradation pathways as we suggest here for Met30. To further test this idea, we measured degradation of the F-box proteins Ctf13 and Cdc4 in *skp1-td* mutants (Fig 7A and 7B). Consistent with a previous report [42] Ctf13 degradation was accelerated in *skp1-td* mutants (Fig 7A). Cdc4 is mainly regulated via autoubiquitylation. In *skp1-td* mutants, Cdc4 was slightly more stable compared to wild type cells, but still degraded rapidly even though Skp1 was depleted as evident by deubiquitylated Met4 (Fig 7B) and elongated, multibudded cells. Rapid degradation of Cdc4 even in the absence of Skp1 is inconsistent with previous reports showing that deletion of the F-box region in Cdc4 significantly stabilizes the protein [11]. However, in the Cdc4^ΔFbox^ mutant used in this study the F-box deletion extended into the region corresponding to the degron region in Met30 [43] providing a possible explanation for these conflicting results.

If the autocatalytic pathway was the major mode of degradation responsible for maintaining F-box protein homeostasis then reduction in Skp1 levels should induce complete stabilization of these proteins. Thus, these results provide evidence for existence of an additional, Skp1-independent, degradation pathway for several F-box proteins, which appears to target F-box proteins that are not bound to the SCF core complex. We refer to this degradation pathway as ‘Skp1-Free’ F-box protein degradation pathway to demarcate it from the autoubiquitylation mode of F-box protein regulation.

### ‘Skp1-free’ F-box degradation is important for cellular function

**Fig 8:**
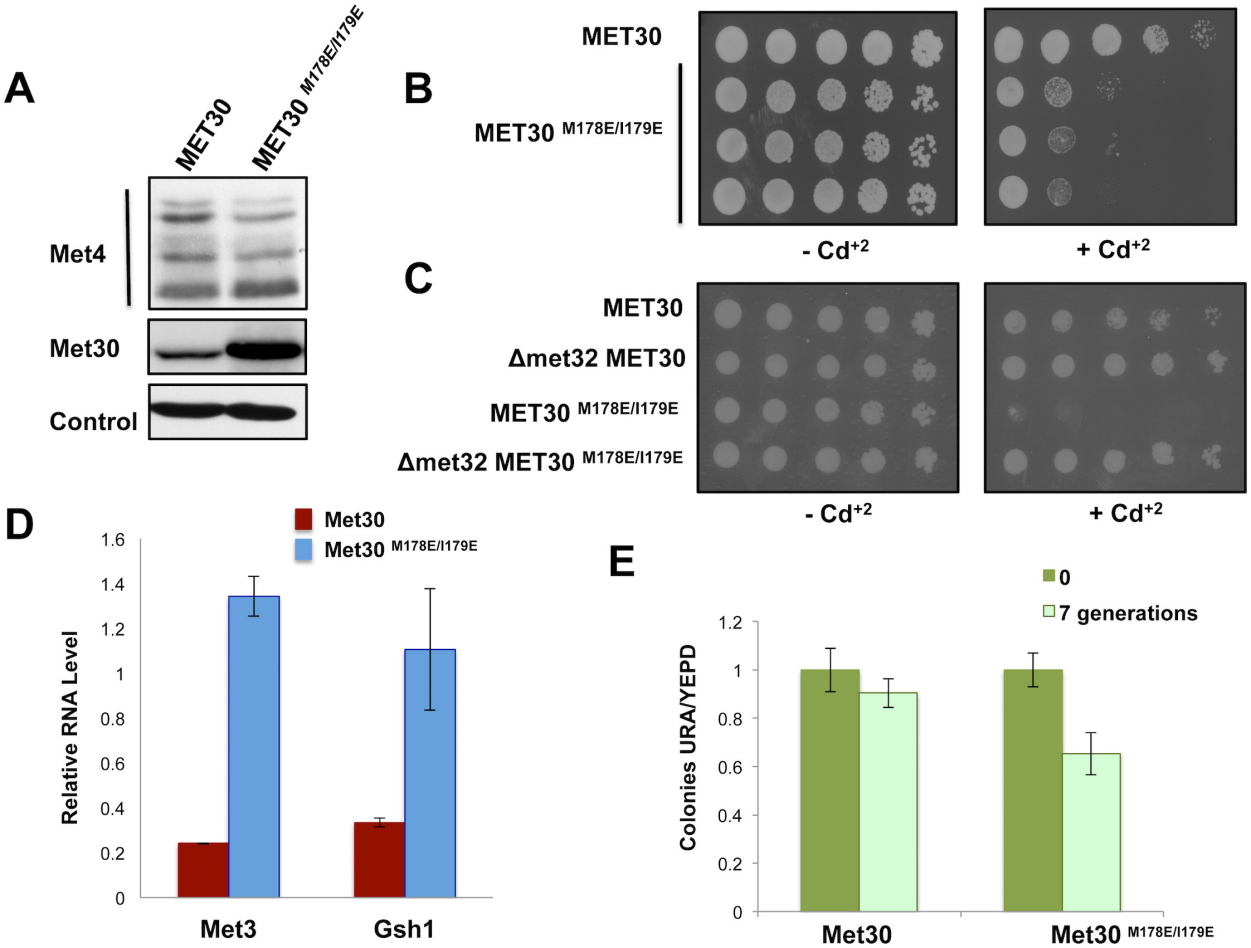
‘Skp1-free’ Met30 degradation is important for cellular functions. (A) Steady state protein levels for Met30 and Met30^M178E/I179E^ strains were compared by immunoblotting. Anti-myc and anti-Met4 antibodies were used for detection of Met30 and Met4, respectively. Cim5 was used as loading control. (B) Met30 and Met30^M178E/I179E^ strains were grown at 30°C to mid-log phase and serial dilutions of cells were spotted on YEPD plates with or without 50μM CdCl_2_. Plates were incubated at 30°C for 2 days. (C) Experiment performed as in panel B, except indicated strains were spotted on minimal medium (SC-LEU) plates with or without 25μM CdCl_2_. Different cadmium concentrations were used because cadmium sensitivity is dependent on media conditions [24] (D). Derepression of Met4 target genes in Met30^M178E/I179E^ mutant. Met30 and Met30^M178E/I179E^ strains were cultured in YEPD. Expression of the Met4 target genes, *MET3* and *GSH1* was analyzed by rt-qPCR (n = 3). Data are represented as mean ± SD. (E) Met30 and Met30^M178E/I179E^ strains containing centromeric plasmid (YCpURA) were grown with and without selection in SC-URA and YPD media respectively. Equal number of cells were counted and plated on SC-URA and YPD plates for both conditions. Plates were incubated at 30°C for 2 days. Plasmid stability was determined by counting number of colonies on SC-URA and YPD plates. Data are represented as mean ± SD (n = 3).

To explore the significance of the ‘Skp1-free’ F-box protein degradation pathway in normal cellular dynamics, we generated yeast strains bearing either wild type Met30 or the Met30^M178E/I179E^ mutant, each controlled by the native *MET30* promoter. As expected, Met30 displayed increased abundance in cells expressing the degron point mutant in comparison to those expressing the wild type allele (Fig 8A). Interestingly, despite the increased protein abundance of Met30^M178E/I179E^ steady-state ubiquitylation of the major SCF^Met30^ substrate, the transcription factor Met4, [25], was slightly reduced (Fig 8A). The M178E/I179E double mutant specifically blocks Skp1-independent degradation of Met30 and it is thus conceivable that Met30 not bound to Skp1, which is normally rapidly degraded, is particularly increased in the Met30^M178E/I179E^ strain. Consequently, a fraction of Met4 could be protected from ubiquitylation if it interacts with excess Met30^M178E/I179E^ that cannot find a Skp1 binding partner.

We reasoned that the Skp1-independent degradation pathway should be particularly important for the recovery from heavy metal stress. Cadmium exposure leads to dissociation of Met30 from Skp1 [24,26,27] thereby generating a burst of ‘Skp1-free’ Met30, which needs to be controlled by the ‘Skp1-free’ F-box protein degradation pathway. We therefore tested cadmium sensitivity of wild type and *MET30* ^*M178E/I179E*^ strains. Cells expressing Met30^M178E/I179E^ were significantly more sensitive and exhibited a growth arrest in response to cadmium stress (Fig 8B). We hypothesized that excess dissociated Met30 may bind Met4 and shield it from ubiquitylation by fully assembled SCF^Met30^ ligase during the recovery phase. The cell cycle checkpoint arrest, initially induced by deubiquitylated Met4 to cope with cadmium stress, may therefore be erroneously maintained in *MET30* ^*M178E/I179E*^ mutants resulting in apparent cadmium sensitivity. The observed growth defect in the presence of cadmium may not indicate a failure to detoxify cadmium but a defect in reversing cell cycle arrest. We tested this hypothesis by deleting *MET32* in *MET30* ^*M178E/I179E*^ mutants (Fig 8C). Met32 is essential for execution of the Met4-induced cell cycle arrest, but its transcriptional role, which is important for cadmium detoxification, is redundant with Met31 [24,26]. In accordance with our hypothesis, deletion of *MET32* suppressed the growth defect of cells expressing Met30^M178E/I179E^ under cadmium stress (Fig 8C), indicating that excess dissociated Met30^M178E/I179E^ interferes with timely inactivation of Met4. Consistent with this idea, two tested Met4 target genes, *MET3* and *GSH1*, were derepressed in Met30^M178E/I179E^ mutants confirming untimely Met4 activation (Fig 8D). Together, these results indicate that the ‘Skp1-free’ F-box protein degradation pathway plays an important role in cellular function to prevent substrate shielding effects by excess unbound F-box proteins.

In addition to enhanced cadmium sensitivity, Met30^M178E/I179E^ mutants displayed increased chromosome loss (Fig 8E). The mechanism for this defect is not known. Protection of an unknown substrate in analogy to Met4 shielding during recovery from cadmium stress is a possible mechanism. However, it is also conceivable that stabilized Met30^M178E/I179E^ interferes locally with Skp1 functions in kinetochore assembly [42,44]

## Discussion

We describe a novel pathway for regulation of F-box protein abundance in addition to autoubiquitylation that specifically targets F-box proteins that are dissociated from Skp1. The architectural theme of SCF ubiquitin ligases employs multiple F-box proteins that bind a common Skp1/Cdc53/Rbx1 core. This arrangement is effective in providing an array of diverse ubiquitin ligases. However, the modular design presents cells with the challenge of balancing the diversity and abundance of different SCF complexes when many F-box proteins, in the case of plants several hundred [45], compete for the shared SCF core components. Cycles of cullin neddylation and CAND1 association maintain a critical level of unoccupied SCF core complexes [9,12,46] while Skp1- F-box protein heterodimers are displaced to bind substrates and recruit them to the Cdc53/Rbx1 complex [47]. This CAND1/Nedd8 cycle maintains dynamic exchange of substrate adapters, but abundance of individual SCF ligases and overall SCF diversity is dictated by the distribution of F-box protein concentrations. It is thus critical to regulate F-box protein levels. In this study we characterize a ‘Skp1-free’ F-box protein degradation pathway that plays an important role in maintaining F-box protein homeostasis. F-box proteins that associate with Skp1 form functional ligases while those that do not, are recognized by the ‘Skp1-free’ F-box protein degradation pathway and degraded by the Cdc53/Rbx1 ligase thereby preventing competition between F-box proteins, limiting substrate shielding effects and ensuring representation even of low abundance F-box proteins in the cellular SCF repertoire. Degradation of F-box proteins that are not bound to Skp1 may also provide an important quality control mechanism to remove damaged F-box proteins. Such a mechanism may be critical for cells because F-box proteins incapable of forming active SCF ligases could maintain an intact substrate binding domain and thus shield their substrates from degradation.

We describe the ‘Skp1-free’ F-box protein degradation pathway in detail for Met30, the substrate adaptor for SCF^Met30^ ubiquitin ligase, which negatively regulates transcription factor Met4 by proteolysis-independent ubiquitylation [23,48,49]. The ‘Skp1-free’ F-box protein degradation pathway is of particular importance for Met30. First, because Met4 ubiquitylation does not induce its degradation under normal growth conditions, Met4 remains associated with Met30 and therefore prevents the canonical F-box-protein degradation pathway through autoubiquitylation. Accordingly, we observed that the ‘Skp1-free’ degradation pathway is the predominant pathway that ensures turn over of excess Met30 (Fig 5C). Second, heavy metal stress induces active dissociation of Met30 from Skp1 resulting in a burst of ‘Skp1-free’ Met30, which interferes with recovery from cadmium stress when the ‘Skp1-free’ degradation pathway is blocked (Fig 8B and 8C).

Indications for a degradation pathway that targets F-box proteins that are not bound to Skp1 have been reported previously [14,42]. In addition, we show that apart from Met30, the two other essential yeast F-box proteins—Cdc4 and Ctf13 are also degraded in the absence of Skp1 (Fig 7A and 7B), suggesting that this pathway is a common mechanism to restrict F-box protein abundance outside the SCF complex. However, not all F-box proteins are subject to this degradation mechanism, because consistent with other reports [10] we found that deletion of the F-box region in Grr1 or inactivation of Skp1 stabilized Grr1 (S5 Fig). The F-box protein degradation pathway we describe here is thus not universal and is probably functional for only those F-box proteins, which harbor the hydrophobic region adjacent to the F-box domain, which is paramount for Cdc53/Rbx1 ligase binding.

In addition to advancing understanding of SCF ligase regulation, our results also demonstrate a function for Cdc53 (cullin-1) independent from its adaptor Skp1. Not only was Met30 degradation active in the absence of Skp1 *in vivo* (Fig 3C), but in addition a Cdc53 mutant incapable of binding to Skp1 could fully complement the Met30 degradation defect of *cdc53* mutants (Fig 4). In addition, Cdc53/Rbx1 can directly bind Met30 in the absence of Skp1 *in vitro* and binding depends on the degron region adjacent to the F-box motif (Fig 6). However, Met30 ubiquitylation in this minimal *in vitro* system with Cdc53/Rbx1, Cdc34, and E1 was very inefficient and required a long reaction time suggesting possible involvement of additional factors, inefficient protein folding in *E*. *coli*, or a post-translational modification lacking in this expression system. Further studies need to be conducted to explore these options in detail.

The findings reported here illustrate a Skp1 independent function for the cullin Cdc53 in substrate ubiquitylation. Consistent with these results, a Skp1 independent cullin-1 based ubiquitylation event has been suggested previously in human cells where Rictor, a component of mTORC2 complex associates with Cullin-1 instead of Skp1, to form a functional E3 ubiquitin ligase that promotes ubiquitylation of SGK1 [50].

Together, our findings shed light on the regulation and complexity of E3 ligases and suggest additional diversity in the cullin-RING family of ubiquitin ligases.

## Materials and Methods

### Yeast strains and growth conditions

Yeast strains used in this study are isogenic to 15DaubΔ, ***a*** *bar1*Δ *ura3*Δns; a derivative of BF264-15D [51] and are listed in S1 Table. Standard culture media and yeast genetic techniques were employed [52]. Determination of protein degradation rates was done using cycloheximide chase experiments and galactose shut- off experiments. For cycloheximide chase experiments, strains carrying plasmids expressing tagged genes of interest placed under their endogenous promoter were cultured to logarithmic phase and cycloheximide (final concentration 100 μg/ml) was added and cells were collected at time points as indicated. For galactose shut-off experiments, strains expressing genes of interest under the control of *GAL1* promoter were cultured in media containing 2% sucrose to logarithmic phase and cultures were transferred to rich media containing 2% galactose (YEPG) for 2 hours or as otherwise indicated. To terminate expression from the *GAL1* promoter cells were transferred to YEPD and collected at the indicated time intervals. Quantitation of protein levels was performed using a Fuji LAS-4000 imaging system followed by analyses with the Multi Gauge v3 software.

### Protein analysis

For immunoblot analysis, yeast whole cell lysates were prepared under denaturing conditions in urea buffer and for immunoprecipitation cells were lysed in Triton X-100 Buffer, as previously described [53]. For purification of HBTH-tagged Met30^ΔFbox^, cells were lysed and purified under denaturing conditions in binding buffer (8M urea, 300mM NaCl, 0.5% NP-40, 50mM PO_4_ pH 8, 50mM Tris-HCl pH 8, 20mM imidazole). 1 mg of total protein lysates was used for binding to Ni^2+^-sepharose (GE Healthcare). Beads were then washed 3 times in binding buffer (without imidazole and pH adjusted to 6.3) and eluted in 150μl elution buffer (8M urea, 200mM NaCl, 50mM PO4, 2% SDS, 10mM EDTA, 100mM Tris-HCl, pH 4.3).

For immunoblot analyses proteins were separated by SDS-PAGE and transferred to a polyvinylidene difluoride membrane. Proteins were detected with the following primary antibodies: anti-Met4 (1:10000; a gift from M. Tyers), anti-Skp1 (1:5000; a gift from R. Deshaies), anti-myc and anti-HA (1:2000; Covance, Princeton, NJ), anti-RGS6H (1:2000; QIAGEN, Germantown, MD), anti-ubiquitin (1:2000; P4G7, #sc-53509, Santa Cruz), anti-Cdc53 (1:1000; yN-18, #sc-6716, Santa Cruz) and anti-MBP (1:2000; N-17, #sc-809, Santa Cruz).

### Purification of recombinant proteins

Plasmids expressing MBP- Met30^(1–186)^ and MBP- Met30^(1–186)M178E/I179E^ were cloned by standard techniques into the pET28 vector and transformed into Rosetta cells. Cells were grown at 37°C and induced with 0.5 mM IPTG for 3 hours. Cells were collected, washed once with cold water, pelleted and frozen. Pellets were later suspended in recombinant protein buffer (0.05% Triton-X100, 0.05% NP-40, 150 mM NaCl, 1 mM PMSF, 1 μg/ml each aprotenin, leupeptin and pepstatin), sonicated and cleared by centrifugation at 13,000 rpm at 4°C for 10 minutes. Lysates were bound to prewashed amylose beads (New England Biolabs) for 3 hours at 4°C. Beads were then washed twice with lysis buffer and thrice with buffer U (50 mM Tris pH 8, 50 mM NaCl, 5 mM ATP, 10 mM MgCl_2_, 0.2 mM DTT). MBP-tagged proteins were eluted in buffer U supplemented with 10 mM maltose. Purified protein was concentrated using Amicon Ultra 50 kDa centrifugal filter and flash frozen.

### *In vitro* binding assay

For *in vitro* binding assay with yeast lysates, yeast cells containing a *GAL1-controlled SKP1* allele and expressing endogenous TAP-tagged Cdc53 were grown in YEP galactose overnight, washed and then transferred to YEP dextrose media for 12 hours to deplete Skp1 protein levels. At this point cells were arrested and showed the characteristic elongated buds. MBP, (MBP)-Met30^(1–186)^ and (MBP)-Met30^(1–186)M178E/I179E^ were lysed and bound to amylose resin as described above. Yeast cell pellets were also lysed in recombinant protein buffer and 3 mg of lysate was incubated with MBP tagged proteins bound to amylose resin, for 2 hours at 4°C. Beads were washed thrice with recombinant protein buffer and bound proteins were eluted by boiling beads in 2x SDS loading buffer.

For the *in vitro* binding experiment performed with Cdc53/ ^GST^Rbx1 expressed from bacteria, bacterial cell pellets were lysed in recombinant protein buffer, sonicated and 3 mg of lysate was incubated with MBP tagged proteins conjugated to amylose resin, for 2 hours at 4°C. Beads were washed thrice with recombinant protein buffer and bound proteins were eluted by boiling beads in 2x SDS loading buffer.

### Real time PCR

RNA samples were isolated, and analyzed by real- time Reverse Transcriptase (RT)-PCR as described [53]. Three biological replicates were analyzed for each experiment.

### Plasmid stability assay

Strains harboring a centromeric plasmid with a *URA3* selection marker were grown at 30°C in minimal medium lacking uracil to force cells to maintain the centromeric plasmid. 200 cells were plated on YPD and minimal media (SC-URA) plates. The remaining cells were cultured without selection for 22 hours (~ 7 generations). Cells were counted and 200 cells were plated again on YPD and minimal media plates to measure the number of cells that have lost the centromeric plasmid. Plates were incubated for 2 days at 30°C and chromosome/plasmid loss was determined by difference in number of colonies on SC-URA plates and YPD plates. Experiments were performed in triplicates.

## Supporting Information

S1 Fig Conditions that disrupt Met30-Skp1 binding destabilize Met30. (A) Cells expressing ^RGS6H^Met30^ΔFbox^ under control of *GAL1* promoter was grown in sucrose medium at permissive temperature at 30°C. Expression of Met30 was induced by addition of 2% galactose for 2.5 h following which 2% dextrose was added to repress *GAL1-MET30* expression. Samples were collected at the time intervals indicated and analyzed by immunoblotting with anti-RGS6H antibodies. (B) Cells expressing ^12Myc^Met30 and ^12Myc^Met30^L187D^ were grown at 30°C. ^12Myc^Met30 was immunopurified and co-purified proteins were analyzed by immunoblotting. A yeast strain expressing untagged Met30 was used as a control. WCE: Whole cell extract (C) Cells expressing ^RGS6H^Met30 under control of *GAL1* promoter were grown in sucrose medium at permissive temperature (25°C). Expression of Met30 was induced by addition of 2% galactose for 1 h, cells were shifted to 37°C for 1.5 h to inactivate the temperature sensitive allele, and 2% dextrose was added to repress *GAL1-MET30* expression. Cadmium was added to a final concentration of 200μM. Samples were collected at the time intervals indicated and analyzed by immunoblotting with anti-RGS6H antibodies.
(TIF)

S2 Fig (A and B) Degradation of ‘Skp1-Free’ Met30 is not dependent on Met4 and Lag2. Cycloheximide chase experiment as described for Fig 1B, was performed in wild type, *MET4* deleted and *LAG2* deleted cells and ^12myc^Met30^ΔFbox^ stability was assayed.
(TIF)

S3 Fig **(A) Mutations in residues important for dimerization domain of Met30 are not essential for the ‘Skp1-free’ Met30 degradation pathway**. Cells expressing either endogenous ^12myc^Met30^ΔFbox^ or different Met30^ΔFbox^ deletion mutants were grown at 30°C. Protein translation was inhibited by addition of cycloheximide and cells were collected at the time intervals indicated. Met30^ΔFbox^ stability was analyzed by immunoblotting with anti-myc antibodies. (B) Smaller deletions within Met30^ΔFbox^ suggesting that the degron for the ‘Skp1-free’ Met30 degradation pathway lies within 170–187 amino acids of Met30. Experiment same as for panel S3A.
(TIF)

S4 Fig Cdc53/Rbx1 can ubiquitylate Met30 *in vitro*. (A) (MBP)-Met30^(1–186)^ was immobilized to amylose resin and incubated with Cdc53^267-851^/Rbx1 expressed in bacteria. Substrate-ligase complex was eluted with 10mM maltose and incubated with ubiquitylation reaction mix for 16 h at 30°C. Ubiquitylation was analyzed by immunoblotting with anti-MBP antibody. (B). Cdc53^267-851^/Rbx1 was purified on glutathione sepharose beads. Efficiency of purification was determined by immunoblotting the eluate with anti-Cdc53 antibody and anti-GST antibody (top panel). In vitro ubiquitylation reaction was performed with purified (MBP)-Met30^(1–186)^ and (MBP)-Met30^(1–186)M178E/I179E^ and purified Cdc53^267-851^/Rbx1. ‘0’ and 16 h time points were collected. Ubiquitylation profile was assayed by immunoblotting with anti-MBP antibody (bottom panel).
(TIF)

S5 Fig Grr1 is not regulated via ‘Skp1-free’ F-box protein degradation pathway. (A). *GAL1* promoter shut off experiment as described in Fig 1C, but experiment was performed with cells expressing either endogenous ^3Myc^Grr1 or ^3myc^Grr1^ΔFbox^ (residues 320–360 deleted). (B). Experiment as in panel A, but ^3myc^Grr1^ΔFbox^ stability was analyzed in wild type and *skp1-25* temperature sensitive mutants.
(TIF)

S1 Table Yeast strains used in this study. (DOCX)
