# Supplementary figures and images for: Skp1 Independent Function of Cdc53/Cul1 in F-box Protein Homeostasis
Source: PLoS Genet. 2015 Dec 10;11(12):e1005727. doi: 10.1371/journal.pgen.1005727 (PMC4675558; doi:10.1371/journal.pgen.1005727)

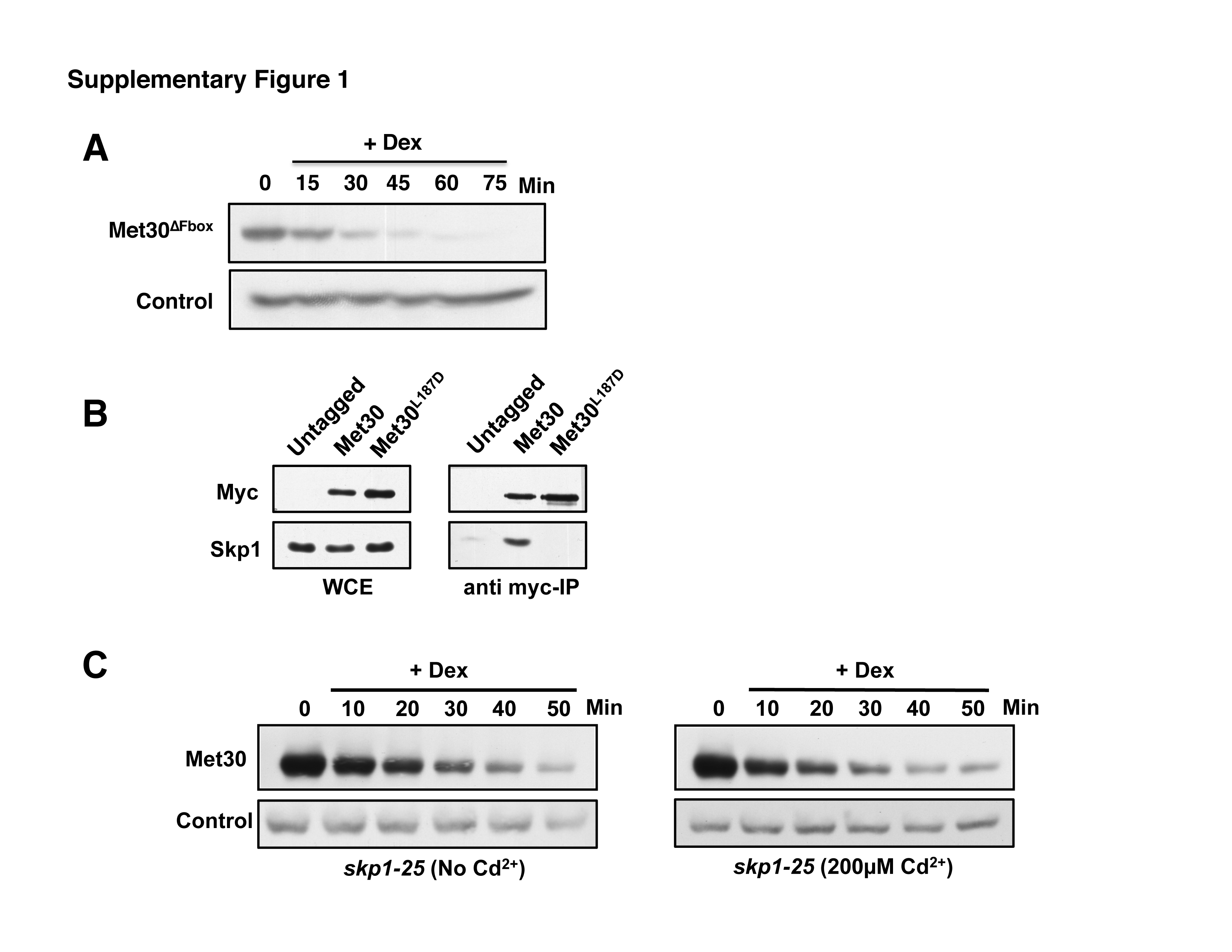

Supplement: S1 Fig — (A) Cells expressing RGS6HMet30ΔFbox under control of GAL1 promoter was grown in sucrose medium at permissive temperature at 30°C. Expression of Met30 was induced by addition of 2% galactose for 2.5 h following which 2% dextrose was added to repress GAL1-MET30 expression. Samples were collected at the time intervals indicated and analyzed by immunoblotting with anti-RGS6H antibodies. (B) Cells expressing 12MycMet30 and 12MycMet30L187D were grown at 30°C. 12MycMet30 was immunopurified and co-purified proteins were analyzed by immunoblotting. A yeast strain expressing untagged Met30 was used as a control. WCE: Whole cell extract (C) Cells expressing RGS6HMet30 under control of GAL1 promoter were grown in sucrose medium at permissive temperature (25°C). Expression of Met30 was induced by addition of 2% galactose for 1 h, cells were shifted to 37°C for 1.5 h to inactivate the temperature sensitive allele, and 2% dextrose was added to repress GAL1-MET30 expression. Cadmium was added to a final concentration of 200μM. Samples were collected at the time intervals indicated and analyzed by immunoblotting with anti-RGS6H antibodies. (TIF) [file pgen.1005727.s001.tif]

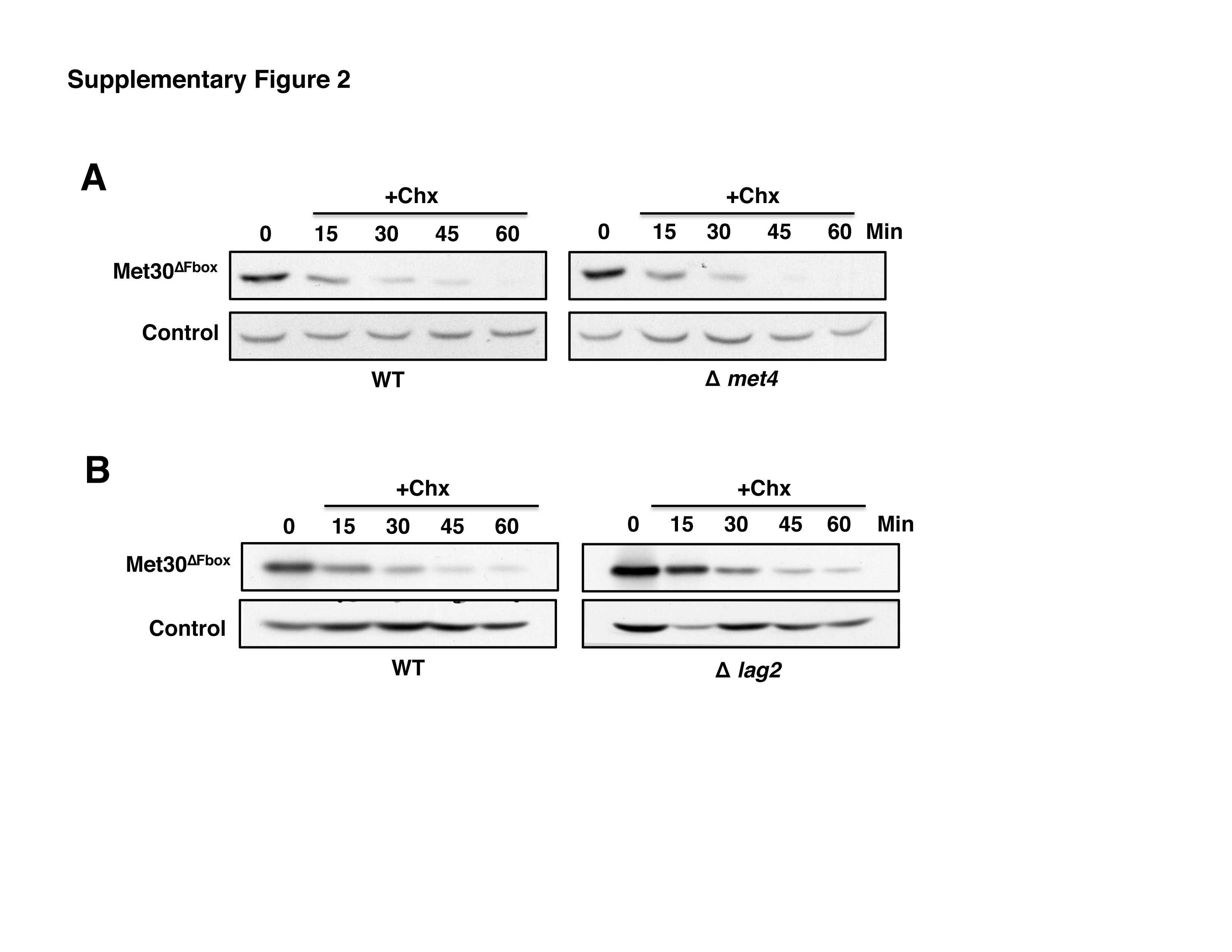

Supplement: S2 Fig — Cycloheximide chase experiment as described for Fig 1B, was performed in wild type, MET4 deleted and LAG2 deleted cells and 12mycMet30ΔFbox stability was assayed. (TIF) [file pgen.1005727.s002.tif]

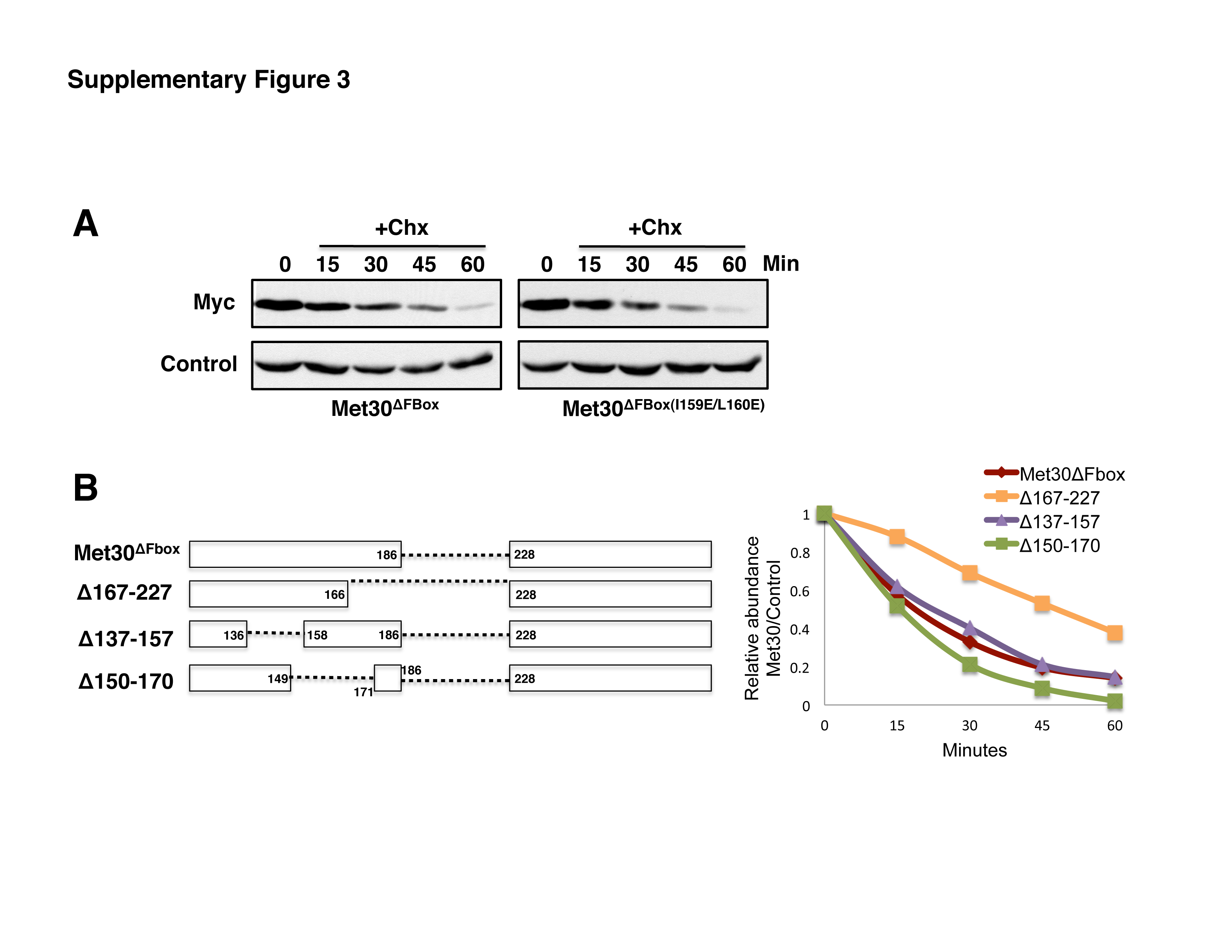

Supplement: S3 Fig — (A) Mutations in residues important for dimerization domain of Met30 are not essential for the ‘Skp1-free’ Met30 degradation pathway. Cells expressing either endogenous 12mycMet30ΔFbox or different Met30ΔFbox deletion mutants were grown at 30°C. Protein translation was inhibited by addition of cycloheximide and cells were collected at the time intervals indicated. Met30ΔFbox stability was analyzed by immunoblotting with anti-myc antibodies. (B) Smaller deletions within Met30ΔFbox suggesting that the degron for the ‘Skp1-free’ Met30 degradation pathway lies within 170–187 amino acids of Met30. Experiment same as for panel S3A. (TIF) [file pgen.1005727.s003.tif]

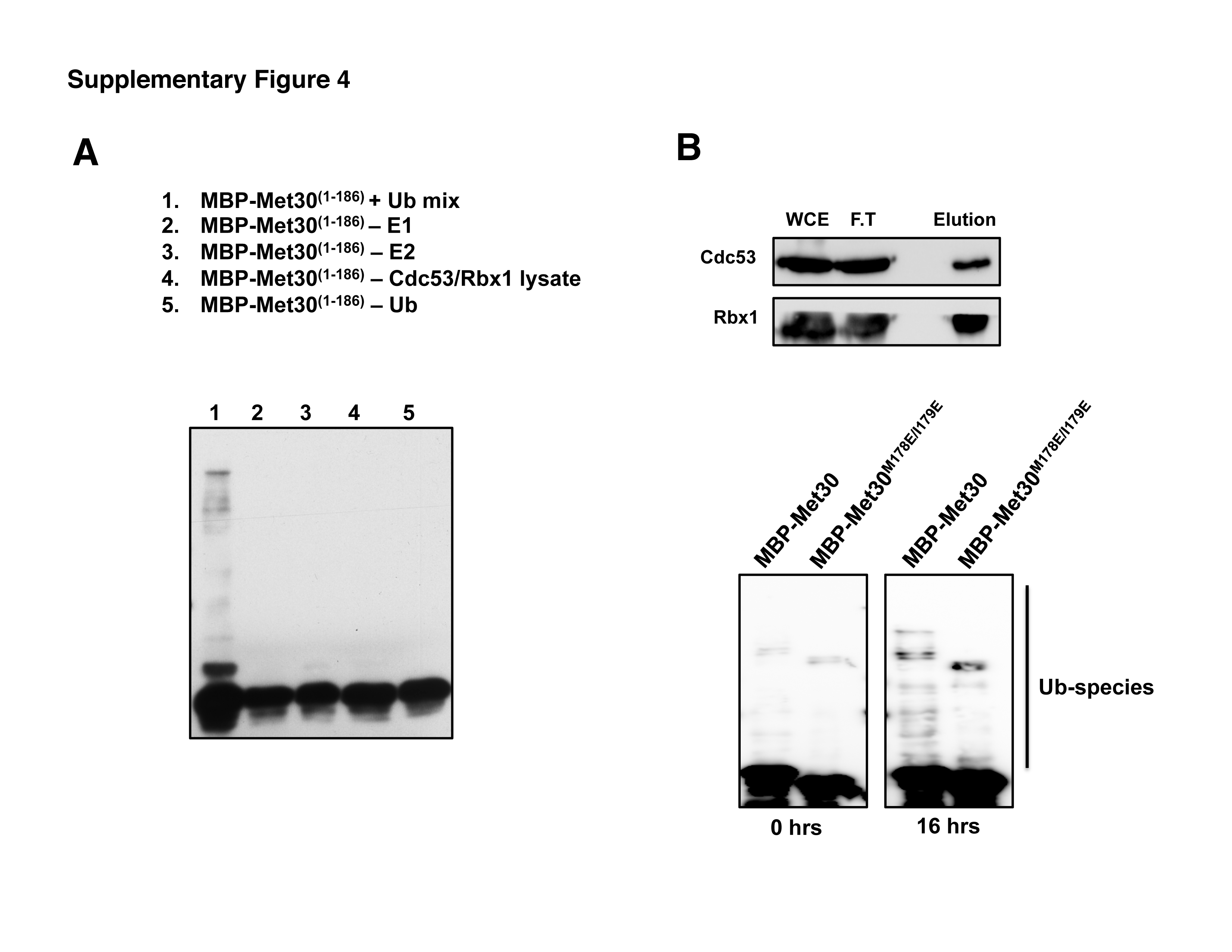

Supplement: S4 Fig — (A) (MBP)-Met30(1–186) was immobilized to amylose resin and incubated with Cdc53267-851/Rbx1 expressed in bacteria. Substrate-ligase complex was eluted with 10mM maltose and incubated with ubiquitylation reaction mix for 16 h at 30°C. Ubiquitylation was analyzed by immunoblotting with anti-MBP antibody. (B). Cdc53267-851/Rbx1 was purified on glutathione sepharose beads. Efficiency of purification was determined by immunoblotting the eluate with anti-Cdc53 antibody and anti-GST antibody (top panel). In vitro ubiquitylation reaction was performed with purified (MBP)-Met30(1–186) and (MBP)-Met30(1–186)M178E/I179E and purified Cdc53267-851/Rbx1. ‘0’ and 16 h time points were collected. Ubiquitylation profile was assayed by immunoblotting with anti-MBP antibody (bottom panel). (TIF) [file pgen.1005727.s004.tif]

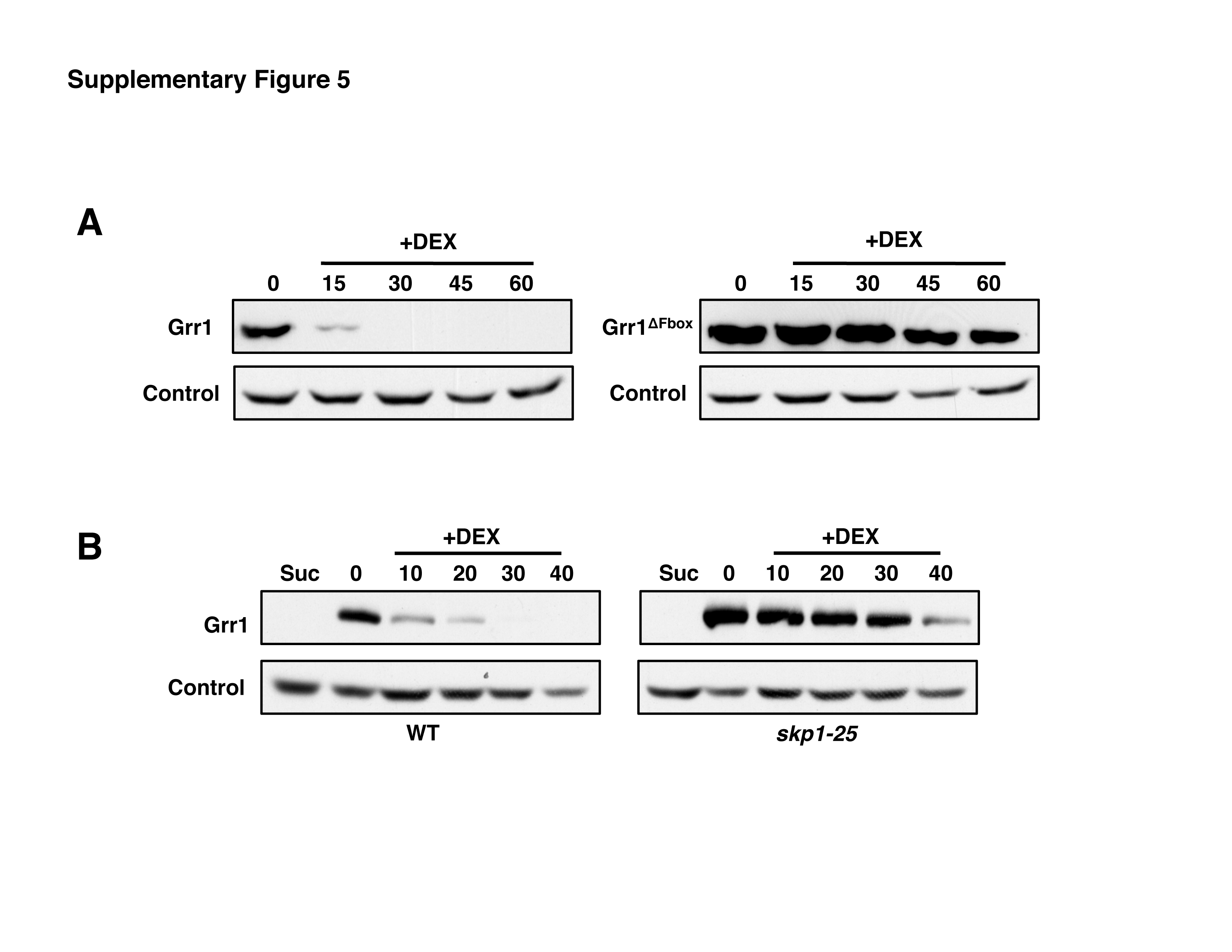

Supplement: S5 Fig — (A). GAL1 promoter shut off experiment as described in Fig 1C, but experiment was performed with cells expressing either endogenous 3MycGrr1 or 3mycGrr1ΔFbox (residues 320–360 deleted). (B). Experiment as in panel A, but 3mycGrr1ΔFbox stability was analyzed in wild type and skp1-25 temperature sensitive mutants. (TIF) [file pgen.1005727.s005.tif]
