# Supplementary material for: Skp1 Independent Function of Cdc53/Cul1 in F-box Protein Homeostasis
Source: PLoS Genet. 2015 Dec 10;11(12):e1005727. doi: 10.1371/journal.pgen.1005727 (PMC4675558; doi:10.1371/journal.pgen.1005727)
Supplement: S1 Table — (DOCX) [file pgen.1005727.s006.docx]

**Table S1:** Strains used in this study

| **Strain** | **Relevant Genotype** |
| --- | --- |
| 15Daub | a *bar1*Δ *ura3*Δ*ns*, *ade1* *his2* *leu2-3112 trp1-1* |
| PY1694 | a *bar1 YCpLeu-13Myc-Met30* |
| PY1695 | a *bar1 YCpLeu-13Myc-Met30(∆187-227)* |
| PY1673 | a *bar1 YCpLeu-GAL1-RGS6His-Met30(∆187-227)* |
| PY1682 | a *bar1 cdc34-3 YCpLeu-GAL1-RGS6His-Met30(∆187-227)* |
| PY1683 | a *bar1 cdc53-1 YCpLeu-GAL1-RGS6His-Met30(∆187-227)* |
| PY1715 | a *bar1 Rbx1-13Myc::KAN YCpLeu-13Myc-Met30(∆187-227)* |
| PY1758 | a *bar1 skp1::KAN YCpTrp-skp1-25 YCpLeu-13Myc-Met30(∆187-227)* |
| PY1759 | a *bar1 cdc53-1 skp1::KAN YCpTrp-skp1-25 YCpLeu-13Myc-Met30(∆187-227)* |
| PY1938 | a *bar1 YCpTrp-GAL1-HBTH-Met30(∆187-227)* |
| PY2196 | a *bar1 skp1::KAN YCpTrp-skp1-25 YCpTrp-GAL1-HBTH-Met30(∆187-227)* |
| PY1941 | a *bar1 cdc53-1 YCpTrp-GAL1-HBTH-Met30(∆187-227)* |
| PY1556 | a *bar1 CUP1-td-3HA-skp1::URA3* |
| PY1705 | a *bar1 CUP1-td-3HA-skp1::URA3 YCpLeu-13Myc-Met30(∆187-227)* |
| PY1742 | a *bar1 CUP1-td-3HA-skp1::URA3 YCpLeu-GAL1-13Myc-Ctf13* |
| PY1798 | a *bar1 CUP1-td-3HA-skp1::URA3 YCpLeu-GAL1-3Myc-Cdc4* |
| PY2146 | a *bar1 met30::HYG met32::KAN YCpLeu-13Myc-Met30(∆137-277)* |
| PY2144 | a *bar1 met30::HYG met32::KAN YCpLeu-13Myc-Met30(∆187-277)* |
| PY2157 | a *bar1 met30::HYG met32::KAN YCpLeu-13Myc-Met30(∆137-227)* |
| PY2210 | a *bar1 met30::HYG met32::KAN YCpLeu-13Myc-Met30(∆187-227)^M178E/I179E^* |
| PY2164 | a *bar1 met30::HYG met32::KAN YCpLeu-13Myc-Met30(∆167-227)* |
| PY2163 | a *bar1 met30::HYG met32::KAN YCpLeu-13Myc-Met30(∆137-157)(∆187-227)* |
| PY2165 | a *bar1 met30::HYG met32::KAN YCpLeu-13Myc-Met30(∆150-170)(∆187-227)* |
| PY2209 | a *bar1 met30::HYG met32::KAN YCpLeu-13Myc-Met30^M178E/I179E^* |
| PY2014 | a *bar1 YCpLeu-GAL1-RGS6His-Met30^L187D^* |
| PY2204 | a *bar1 met4::KAN YCpLeu-13Myc-Met30(∆187-227)* |
| PY2234 | a *bar1 cdc53-1 YCpLeu-13Myc-Met30(∆187-227) YCp-URA-GAL1* |
| PY2247 | a *bar1 cdc53-1 YCpLeu-13Myc-Met30(∆187-227) YCp-URA-GAL1-Cdc53* |
| PY2249 | a *bar1 cdc53-1 YCpLeu-13Myc-Met30(∆187-227) YCp-URA-GAL1-Cdc53^Y133R^* |
| PY1990 | a *bar1 skp1::KAN pep4::URA YIpG2-Skp1 Cdc53-TAP::KAN* |
| PY2240 | a *bar1 met30::HYG YCpLeu-13Myc-Met30* |
| PY2241 | a *bar1 met30::HYG YCpLeu-13Myc-Met30^M178E/I179E^* |
| PY2079 | *a bar1 lag2::KAN YCpLeu-13Myc-Met30(∆187-227)* |
